# ﻿First record of *Lepidiella* Enderlein, 1937 from the Oriental Region (Diptera, Psychodidae)

**DOI:** 10.3897/zookeys.1115.81668

**Published:** 2022-07-29

**Authors:** Santiago Jaume-Schinkel, Gunnar Mikalsen Kvifte

**Affiliations:** 1 Zoologisches Forschungsmuseum Alexander Koenig, Leibniz-Institut zur Analyse des Biodiversitätswandels, Adenauerallee 160, D-53113 Bonn, Germany Zoologisches Forschungsmuseum Alexander Koenig Bonn Germany; 2 Department of Biosciences and Aquaculture, Nord University, P.O. Box 2501, 7729 Steinkjer, Norway Nord University Steinkjer Norway

**Keywords:** Moth flies, new record, new species, Psychodinae, taxonomy

## Abstract

We provide the first record of the genus *Lepidiella* Enderlein, 1937 from the Oriental Region with the description of *Lepidiellalimicornis***sp. nov.**, based on two male specimens collected in Thailand. Additionally, we provide a list of the world species of *Lepidiella* and discuss the diagnosis and taxonomic placement of the genus.

## ﻿Introduction

The moth fly fauna of the Oriental Region is highly diverse and understudied, with the family Psychodidae including more than 420 described species (Lewis 1987; Ježek 2010; Curler and Priyadarsanan 2015; Ježek et al. 2015; Kvifte and Andersen 2016; Polseela et al. 2019). Regardless of the recent attention this family has received due to the medical importance of the subfamily Phlebotominae, there is still a large number of species that remain undescribed (Duckhouse and Duckhouse 2000; Curler 2009; Ježek 2010).

The genus *Lepidiella* Enderlein, 1937, formerly known as *Syntomoza* Enderlein, 1937 (see Quate, 1963; Collantes and Hodkinson 2003) has been thought to be restricted to the Neotropical Region. This genus has been recorded in Brazil, Bolivia, Colombia, Costa Rica, Mexico, Nicaragua, Panama, Peru, and the island of Santa Lucia in the Caribbean (Ibáñez-Bernal 2008; Bravo and Santos 2011; Araújo and Bravo 2013, 2019).

Here, we describe a new species of the genus *Lepidiella* and discuss its generic placement. Additionally, we record *Lepidiella* for the first time outside the Neotropical Region, and we update the generic diagnosis of this genus.

## ﻿Materials and methods

The studied specimens are deposited at the Department of Natural History, University Museum of Bergen, Bergen, Norway (**ZMBN**). Specimens were collected with a hand net, stored in ethanol, and then mounted on permanent slides. In the material examined section, at the end of each record and between square brackets ([]), the holding institution is indicated. In the description of type labels, the contents of each label are enclosed in double quotation marks (“ ”), italics denote handwriting, and the individual lines of data are separated by a double forward-slash (//).

Measurements were made with an ocular micrometer in a microscope Leitz model Dialux 20, measures in millimeters (mm). Head width was taken at the widest part, approximately above the insertion of antennal scape, whereas the length was taken from the vertex to the lower margin of clypeus; wing length measured from the base of the wing at the start of the costal node to the apex of the wingtip, while the width was taken approximately at an imaginary vertical line crossing the radial and medial forks; palpal proportions consider the length of the first palpal segment as a unit (1.0).

### ﻿Terminology

We follow the general terminology proposed by Cumming and Wood (2017). For the male genitalia, we follow the term of hypopods instead of cerci or surstyli proposed by Kvifte and Wagner (2017) as the origin of these caudal appendages seems to have combined origins of the proctiger and epandrium.

## ﻿Results

### ﻿Genus Lepidiella

Taxon classificationAnimaliaDipteraPsychodidae

Enderlein, 1937

4B4D6D44-CA2F-53D0-93F3-C89094AC3EB7

Lepidiella Enderlein 1937: 89. Type species: LepidiellalanuginosaEnderlein 1937: 89–90, by monotypy and original designation.
Syntomoza Enderlein 1937: 88–89. Type species: Syntomoza niveitarsisEnderlein 1937: 89, by monotypy and original designation.
Kupara Rapp 1945: 310. Type species: KuparaalbipedaRapp 1945: 311, by monotypy and original designation (Bravo and Santos 2011; Collantes and Hodkinson 2003).

#### Diagnosis.

Males and females with vertex dorsally expanded; males with or without corniculi, females without corniculi; males and females with 4 rows of facets on eye bridge, antennae with 14 barrel-shaped flagellomeres, flagellomeres 1–11 with a pair of simple digitate ascoids, flagellomeres 12–14 reduced in size and without ascoids; wing vein R_4_ ending slightly before or at the wing apex; males with multiple apical tenacula on hypopods.

#### Species included.

*Lepidiellaalbipeda* (Rapp, 1945), *L.amaliae* (Collantes & Martínez-Ortega, 1997), *L.cervi* (Satchell, 1955), *L.flabellata* Bravo & Santos, 2011, *L.hansoni* (Quate, 1996), *L.lanuginosa* Enderlein, 1937, *L.larryi* Ibáñez-Bernal, 2010, *L.limicornis* sp. nov., *L.maculosa* Araújo & Bravo, 2019, *L.matagalpensis* (Collantes & Martínez-Ortega, 1988), *L.monteveredica* (Quate, 1996), *L.niveitarsis* (Enderlein, 1937), *L.olgae* Bravo & Araújo, 2013, *L.pickeringi* (Quate, 1999), *L.robusta* Bravo & Santos, 2011, *L.spinosa* Bravo, 2005, *L.wagneri* Araújo & Bravo, 2019, *L.zumbadoi* (Quate, 1999).

### ﻿ Lepidiella limicornis sp. nov.

Taxon classificationAnimaliaDipteraPsychodidae

3E0460D5-5F42-5309-805F-96FDD9FB516A

https://zoobank.org/067E7A52-E761-4849-B781-C52EAF35BACE

Figs 1–5

#### Examined material.

***Holotype***, ♂, slide mounted, . “*Lepidiellalimicornis #m* // *HOLOTYPE* // Thailand: Chiang Mai, // Doi Pui Mong village, // waterfall/pond, // 18.8163°N, 98.8831°E // 9.IV.1991, (hand net) // J. Kjaerandsen leg. // **ZMBN** #:”, [ZMBN], paratype, ♂, slide mounted, same label information [ZMBN].

#### Differential diagnosis.

This species can be easily differentiated from all the species in *Lepidiella* by the combination of the following characters: eyes separated by 4 facet diameters, interocular suture as inverted U, second flagellomere asymmetrical, and hypopods with four tenacula.

#### Type locality.

Thailand, Chiang Mai, Doi Pui Mong village (18.8163°N, 98.8831°E).

#### Description.

Measurements in mm (*n* = 2). Wing length 1.81, width 0.68; head length 0.45, width 0.34; Antennal segments, scape: 0.19, pedicel: 0.07, flagellomere 1: 0.08, flagellomere 2: 0.08, flagellomeres 3–9: 0.06; Palpomeres 1: 0.08, 2: 0.12, 3: 0.12, 4: 0.16.

**Male. *Holotype*. *Head*** 2 × longer than wide, with a pair of 3-branched cornicula, eyes separated by approximately 4 facet diameters; eye bridge with four facet rows; interocular suture as an inverted U, extending towards middle of vertex, a little longer than eye bridge width. Antenna with scape about 4× longer than its width, about 3× length of pedicel, cylindrical, tapered at base, and broadening at apex; first flagellomere cylindrical, symmetrical, about ½ width of scape, second flagellomere asymmetrical with a protuberance on inner margin, subsequent flagellomeres symmetrical, cylindrical, about ½ width of first and second flagellomeres. Total number of flagellomeres unknown as apical flagellomeres are missing in examined specimens; maximum number of flagellomeres = 7. Palps extending to flagellomere 6, palpal proportions, 1.0:1.5:1.5:2.

***Wing*** 2.7 × longer than wide, hyaline except costal cell which is brownish; Sc not reaching C but extending to junction of R_2+3_+R_5_; R_4_ ending at wing apex, CuA reaching wing margin.

***Terminalia*.** Hypandrium narrow, with rounded margin, seems partially fused with gonocoxites; length of gonocoxites 0.60 length of gonostyli, about 2× longer than wide; gonostyli narrow, tapered towards apex, with alveoli in outer basal ⅓; gonocoxal apodemes triangular, medial extension connected to base of aedeagus; aedeagus symmetrical, bifurcated; paramere narrow, well sclerotized; ejaculatory apodeme dorsoventrally flattened, rounded at anterior margin and tapering towards aedeagus; epandrium about same length and width; basal margin concave around entire length, apical margin strongly concave at middle; hypopods about 1.75× length of gonocoxites, narrow with apical margin rounded; 4 apical tenacula on each; tenacula apex rounded, concave; epiproct triangular with apical margin rounded, covered in micropilosity.

**Figures 1–5.**
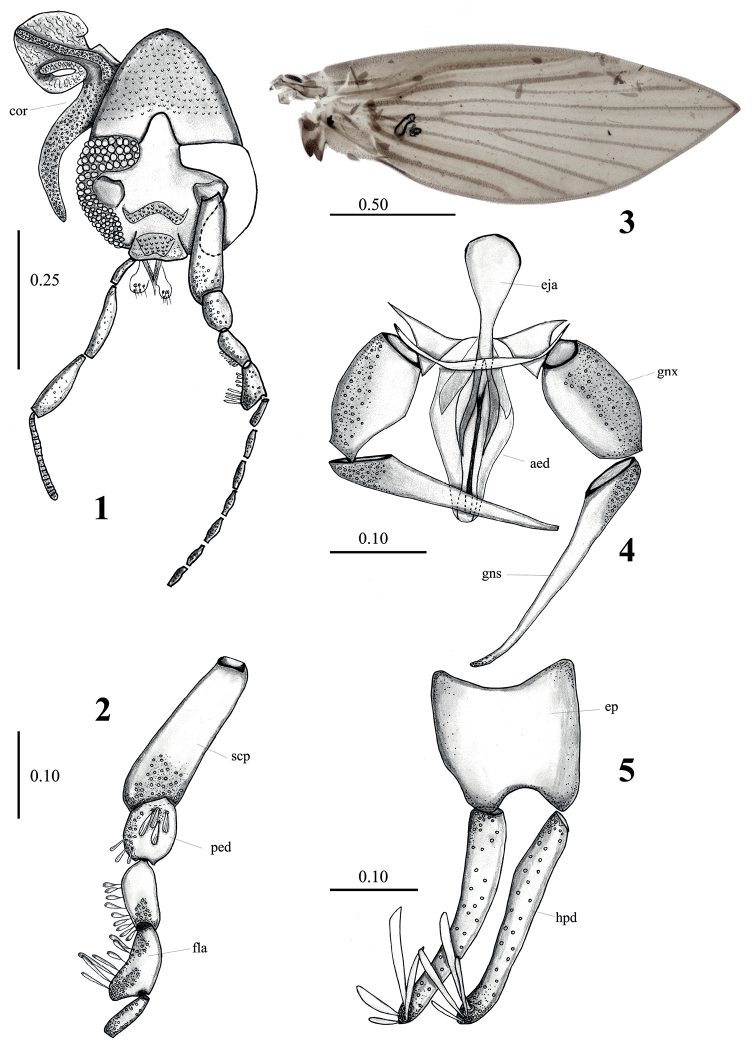
*Lepidiellalimicornis* sp. nov., male holotype. **1** head **2** first antennal segments **3** wing **4** hypandrium, gonocoxites, gonostyli, aedeagus **5** epandrium and surstyli. Abbreviations: aed = aedeagus, cor = corniculi, eja = ejaculatory apodeme, ep = epandrium, fla = flagellomere, gns = gonostylus, gnx = gonocoxites, scp = scape, hpd = hypopod. Scale bars in millimeters.

**Female.** Unknown.

#### Etymology.

From Latin *līmus* = oblique + *cornus* = horns, making references to the oblique shape of the fourth antennal segment (second flagellomere).

#### Distribution.

Only known from the type locality.

## ﻿Discussion

Quate (1996) recognized three diagnostic characters for the genus (as *Syntomoza*): corniculi present in males; males and females with vertex expanded dorsally; males and females with the apex of vein R_4_ ending at the wing apex. Bravo (2005) later described a new species and transferred *Pericomahansoni* (now *Lepidiellahansoni*) without corniculi. Bravo and Santos (2011) updated the diagnosis of the genus. Finally, Araújo and Bravo (2019) described a new species without the presence of corniculi and recognized six characters for the identification of males and females, specifically: vertex dorsally expanded; antenna with 14 barrel-shaped flagellomeres; flagellomeres 12–14 smaller, without ascoids; R4 ending at the wing apex; males with multiple tenacula on hypopods (as cercus); gonocoxal apodemes fused, forming a narrow and plate-like bridge, not extending anteriorly.

Of these six characters only five fit with the species described here: gonocoxal apodemes are not fused and are extended anteriorly. The diagnosis presented above reflects this.

Corniculi are present in many genera, including *Clytocerus* Eaton, 1904, *Jungiella* Vaillant, 1972, *Panimerus* Eaton, 1913, *Pangeogladiella* Ježek, 2001, *Mystropsychoda* Duckhouse, 1975, and *Neoarisemus* Botoseneanu & Vaillant, 1970. However, this species can be easily separated from *Mystropsychoda* by the presence of an eye bridge (absent in *Mystropsychoda*); from *Neoarisemus*, *Panimerus*, and *Jungiella* by barrel-shaped flagellomeres and wing venation with R_4_ ending at the apex of the wing and R_5_ endsing beyond apex (flagellomeres fusiform and R_4_ before and R_5_ at the apex in *Neoarisemus*, *Panimerus*, and *Jungiella*). Finally, *Lepidiella* can be differentiated from *Clytocerus* by the absence of fusion of flagellomeres 1 and 2 (fused in *Clytocerus*), the absence of tuft of curved setae on basal flagellomeres (present in *Clytocerus*), and the setae alveoli of the frons being in a large continuous patch (*Clytocerus* having two separate patches).

The characters separating *Lepidiella* from *Clytocerus* are unique characters for *Clytocerus* and probably represent apomorphies. It may, therefore, be that *Lepidiella* represents either a plesiomorphic sister group to *Clytocerus* or even is the paraphyletic ancestoral taxon to it. As *Clytocerus* generally have fused gonocoxal apodemes, while *Lepidiella* as shown here is polymorphic for this character, we deem it more likely that *Lepidiella* is paraphyletic to *Clytocerus*. However, we refrain from synonymizing the two until more unambiguous characters, including molecular ones, are available.

## Supplementary Material

XML Treatment for Lepidiella

XML Treatment for Lepidiella limicornis
